# Autophagy, innate immunity, and cardiac disease

**DOI:** 10.3389/fcell.2023.1149409

**Published:** 2023-05-10

**Authors:** Donato Santovito, Sabine Steffens, Serena Barachini, Rosalinda Madonna

**Affiliations:** ^1^ Institute for Cardiovascular Prevention (IPEK), Ludwig-Maximilians-University (LMU) Munich, Munich, Germany; ^2^ German Center for Cardiovascular Research (DZHK), Partner Site Munich Heart Alliance, Munich, Germany; ^3^ Unit of Milan, Institute for Genetic and Biomedical Research (IRGB), National Research Council, Milan, Italy; ^4^ Hematology Division, Department of Clinical and Experimental Medicine, University of Pisa, Pisa, Italy; ^5^ Cardiology Division, Cardio-Thoracic and Vascular Department, Azienda Ospedaliero-Universitaria Pisana, Pisa, Italy; ^6^ Department of Surgical, Medical, Molecular Pathology & Critical Care Sciences, University of Pisa, Pisa, Italy

**Keywords:** autophagy, cardiac function, myocardial infarction, immune response, heterophagy, aging, diabetic cardiomiopathy

## Abstract

Autophagy is an evolutionarily conserved mechanism of cell adaptation to metabolic and environmental stress. It mediates the disposal of protein aggregates and dysfunctional organelles, although non-conventional features have recently emerged to broadly extend the pathophysiological relevance of autophagy. In baseline conditions, basal autophagy critically regulates cardiac homeostasis to preserve structural and functional integrity and protect against cell damage and genomic instability occurring with aging. Moreover, autophagy is stimulated by multiple cardiac injuries and contributes to mechanisms of response and remodeling following ischemia, pressure overload, and metabolic stress. Besides cardiac cells, autophagy orchestrates the maturation of neutrophils and other immune cells, influencing their function. In this review, we will discuss the evidence supporting the role of autophagy in cardiac homeostasis, aging, and cardioimmunological response to cardiac injury. Finally, we highlight possible translational perspectives of modulating autophagy for therapeutic purposes to improve the care of patients with acute and chronic cardiac disease.

## 1 Introduction

Adaptation to stress is an arduous challenge for eukaryotic cells. It involves the orchestrated action of multiple pathways to coordinate metabolic activities with nutrient availability, regulate cell cycle and proliferation, and dispose of dysfunctional intracellular elements. These multifaceted programs ultimately direct cell fate toward survival or death, with pathophysiological implications for health and disease (Mizushima and Levine, 2020). Among the major mechanisms involved, autophagy is primarily a catabolic process aimed at recycling intracellular components to maintain nutrient homeostasis, favor metabolic adaptation, prevent damage by dysfunctional organelles, and preserve genomic stability (Levine and Kroemer, 2019; Mizushima and Levine, 2020). While originally considered a non-selective recycling system, we now appreciate autophagy as a complex machine capable of selective target recognition and involved in intracellular and extracellular trafficking of diverse molecules and organelles (Gatica et al., 2018; Kaushik and Cuervo, 2018; Levine and Kroemer, 2019). As such, autophagy has attracted notable scientific interest, as confirmed by > 74,000 related articles and the Nobel prize awarded to Yoshinori Ohsumi in 2016 for the discovery of genes involved in its mechanisms. In parallel, translational research has focused on exploiting autophagy modulation as a potential therapeutic strategy in clinical medicine.

Autophagy is rapidly activated upon stress exposure acting downstream of crucial signaling networks coordinating cell biology with environmental conditions, such as the mTOR, AMPK, GSK-3β, and Hippo pathways (Zhai et al., 2011; Saxton and Sabatini, 2017; Maejima et al., 2022). Multiple cardiovascular-relevant stimuli (e.g., shear stress, hypoxia, ischemia, danger-associated molecular patterns, redox stress, mitochondrial damage) induce autophagy in cell types involved in cardiovascular function (Nakai et al., 2007; Kroemer et al., 2010; Henderson et al., 2021). Hence, it is unsurprising that functional autophagy machinery is required for cardiac function and limits disease development in response to cardiac injuries (e.g., mediating mitochondrial turnover upon hemodynamic overload) (Shirakabe et al., 2016; Miyamoto, 2019). Consistently, suppression of autophagy is a common feature in heart failure, aging, and upon exposure to cardiotoxic drugs (e.g., doxorubicin) (Fernandez et al., 2018; Hahn et al., 2021; Wang et al., 2021). However, excessive activation of autophagy may occur early upon pressure overload or in ischemia/reperfusion injury and might contribute to pathological processes (Matsui et al., 2007; Zhu et al., 2007; Zhang et al., 2021; Nah et al., 2022), thus highlighting the importance of an “activity window” of activation of autophagy. Moreover, autophagy is a ubiquitous process and, besides its biological role in the cardiovascular system, contributes to the maturation and functionality of immune cells (e.g., neutrophils and monocytes) which are crucially involved in response to cardiac injury (Bhattacharya et al., 2015; Germic et al., 2019; Deretic, 2021). In this Review, we will discuss the contribution of autophagy to cardiac pathophysiology, highlighting emerging functional paradigms and their involvement in cells and mechanisms dictating cardiac healing. Finally, we will highlight the opportunity of modulating autophagy for therapeutic purposes in cardiac diseases.

## 2 Autophagy: molecular mechanisms

As its name (Greek for “self-eating”) implies, autophagy is mainly a catabolic process to dispose off intracellular components, typically dysfunctional/senescent organelles or protein aggregates, by proteolytic degradation into lysosomes. Different mechanisms of lysosomal cargo sequestration distinguish three types of autophagy: (i) chaperone-mediated autophagy (CMA), (ii) microautophagy, and (iii) macroautophagy. In CMA, the protein LAMP2A (lysosome-associate membrane protein 2A) recruits into lysosomes the chaperone HSC70 which selectively recognizes cargoes via KFERQ-like motifs (Kaushik and Cuervo, 2018). On the other hand, microautophagy engulfs cytosolic elements in vesicles formed by the invagination of lysosomal membranes (Wang et al., 2022). Finally, macroautophagy involves multistep intracellular membrane rearrangement to seal cargoes into double-membraned vesicles (i.e., autophagosomes) which eventually fuse with lysosomes (Dikic and Elazar, 2018; Levine and Kroemer, 2019). While recent evidence suggests the involvement of CMA in the cardiovascular system (Pedrozo et al., 2013; Qiao et al., 2021; Subramani et al., 2021; Ghosh et al., 2022), the wide core of research has studied the crucial contribution of macroautophagy (commonly referred to as “autophagy”) in mechanisms of cardiac function. Hence, this Review will refer to macroautophagy as “autophagy”, which commonly occurs in literature.

**FIGURE 1:**
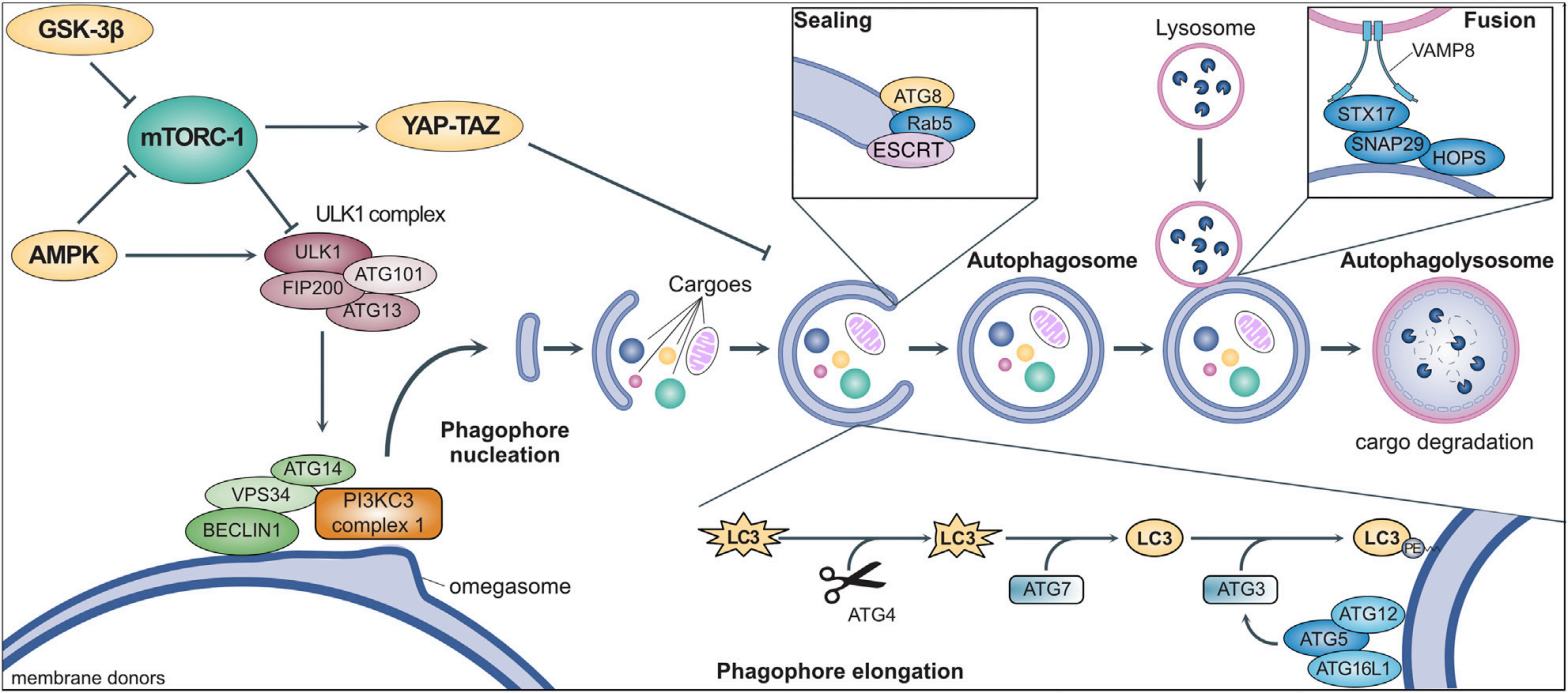
General mechanisms of macroautophagy. Stress signals activate intracellular metabolic pathways (e.g., mTORC-1, GSK-3β, AMPK) that culminate in the regulation of the ULK1 complex. This complex triggers the initiation of autophagy by promoting phagophore nucleation through the activation of the PI3KC3 complex 1 via the phosphorylation of multiple protein, such as BECLIN1, VPS34, and ATG14. After nucleation, the coordinated activity of multiple ATGs mediates phagophore elongation through the conjugation of proteins of the ATG8-family (e.g., LC3). The phagophore engulfs intracellular cargoes (e.g., protein aggregates and dysfunctional organelles) before membrane sealing to produce a double-layered vesicle named autophagosome, the hallmark of macroautophagy. The autophagosome eventually fuses with a lysosome for the degradation of autophagic cargoes, while membraned are recycled and become available for the elongation of other phagophores (see text for more details).

Autophagy involves the coordinated activity of a set of evolutionarily conserved AuTophagy-related Genes (ATGs) (Figure 1) (Yamamoto et al., 2023). It is initiated by the ULK1 (Unc-51-like kinase 1) complex, downstream of multiple signaling pathways involved in nutrient sensing and metabolism (Ganley et al., 2009; Saxton and Sabatini, 2017). Once activated, ULK1 binds to and phosphorylates ATG14, VPS34, and BECLIN1, which translocate to specific sites of the endoplasmic reticulum (named “omegasomes”) and activate the class III PI3K (PI3KC3) complex I (Nishimura et al., 2017). The consequent local production of phosphatidylinositol-3-phosphate (PI3P) starts the nucleation of phagophores, which are precursors of autophagosomes (Hamasaki et al., 2013; Karanasios et al., 2013). Multiple organelles (e.g., mitochondria, Golgi, endosomes) supply membranes for phagophore elongation which are marked by ATG9, the only transmembrane ATG (Orsi et al., 2012). The elongation depends on the ATG8 family members (i.e., LC3 and GABARAP) which are first cleaved by ATG4, then activated by ATG7, and finally anchored to phagophore membranes through conjugation to phosphatidylethanolamine operated by ATG3 (Hamasaki et al., 2013). An efficient ATG8 conjugation requires the activation of ATG3 by the ATG5-ATG12-ATG16L1 complex, which localizes in early phagophores and shapes membrane curvature to facilitate elongation (Jensen et al., 2022). ATG8 conjugation is not permanent and can be reverted by ATG4, resulting in its release from the membrane to limit phagophore elongation (Scherz-Shouval et al., 2007). Indeed, ATG8 promotes elongation upon conjugation, although its requirement has been questioned (Weidberg et al., 2011; Nguyen et al., 2016). The sealing of the phagophore to produce an autophagosome also involves ATG8 and features the contribution of the small GTPase Rab5 and other components of the endosomal sorting complexes required for transport (ESCRT) (Weidberg et al., 2011; Takahashi et al., 2018; Zhou et al., 2019). After being produced, the autophagosome undergoes maturation by removal of ATGs from the outer membrane and recruiting the molecular machinery required for its fusion with lysosomes. This includes the homotypic fusion and protein sorting (HOPS) complex, which mediates membrane tethering (Jiang et al., 2014), and the SNARE receptors STX17 and SNAP29, which bind to ATG14 and are primed for interactions with VAMP8 localized on the lysosomal membrane (Diao et al., 2015). The autophagosome-lysosome fusion encompasses the trafficking of multiple additional proteins (e.g., small GTPases such as Rab7) (Langemeyer et al., 2018) and results in the release of various lysosomal enzymes into the lumen to hydrolyze the cargoes and liberate their molecular building blocks (e.g., amino acids), which are relocated to the cytoplasm for reuse (Yang et al., 2006; Diao et al., 2015). Conversely, the membranes of the autophagosomes are selectively sorted out by an SNX4-SNX5-SNX17 complex licensing their recycling in the intracellular membrane pool (Zhou et al., 2022).

The knowledge of mechanisms of cargo engulfment in phagophore/autophagosomes has sensibly evolved in the last decade. While autophagy was originally branded as a bulk recycling process, the discovery of CMA and its selectivity in target recognition somehow prompted the investigation of mechanisms of selection (Kaushik and Cuervo, 2018). Nowadays, all types of autophagy are recognized as capable of selectivity which is conferred by the binding of ATG8 proteins (e.g., LC3) to cargo receptors, such as proteins SQSTM1 (also known as p62), NBR1, and NIX (also known as BNIP3L) (Gatica et al., 2018). The spatial proximity of these components to the targets allows phagophore elongation around the cargo and its inclusion into nascent autophagosomes. The cargo receptors interact with conjugated LC3 (i.e., LC3-II) through a distinctive and evolutionary conserved WXXL motif named LC3-interacting region (LIR) (Ichimura et al., 2008; Gatica et al., 2018). These receptors mediate biophysical cargo interaction with the phagophore during elongation by recognizing specific “eat me” signals, dependent or independent of ubiquitination (Gatica et al., 2018). Mitophagy is one of the most widely studied forms of selective autophagy to remove dysfunctional mitochondria. Through exposing PINK1 on the outer membrane, damaged mitochondria recruit the E3-ligase PARK2 (also known as Parkin) to ubiquitinylate multiple proteins which are recognized by the cargo receptors optineurin, NDP52, and SQSTM1 (Geisler et al., 2010; Trempe et al., 2013; Lazarou et al., 2015). However, recognition of dysfunctional mitochondria may also occur with ubiquitin-independent pathways via LIRs contained in the proteins NIX, FUNDC1, and BNIP3 (Liu et al., 2012; Ni et al., 2015). Additional examples of selective autophagy include reticulophagy (endoplasmic reticulum), lysophagy (damaged lysosomes), nucleophagy (nucleus), lipophagy (lipid droplets), pexophagy (peroxisomes), aggrephagy (misfolded proteins) (Gatica et al., 2018; Ma et al., 2022). Finally, while organelles and proteins are usually considered, autophagy also targets RNAs either by affecting RNA-binding proteins or by direct interaction of LC3 with RNAs (Gibbings et al., 2012; Liao et al., 2018; Santovito et al., 2020; Hwang et al., 2022; Ma et al., 2022).

Besides their original role of mediating intracellular catabolism, ATGs and autophagy machinery in eukaryotes engage in many other nonconventional functions (Yang et al., 2006; Levine and Kroemer, 2019). Some functions are similar to degradative autophagy, as in the case of LC3-associated phagocytosis which targets extracellular components by engulfing them into single-membraned vesicles marked by LC3 (requiring the ATG8-conjugation machinery) which are directed toward lysosomes (Heckmann and Green, 2019). Yet, the autophagy machinery may direct autophagosomes toward the plasma membrane and promote cargo release (i.e., secretory autophagy), rather than degradation in autophagolysosomes. While molecular mechanisms are still not fully understood, secretory autophagy contributes to the extracellular release of IL-1β, IL-18, and HMGB1 from macrophages (Dupont et al., 2011), to the ejection of dysfunctional mitochondria from cardiomyocytes for their uptake and disposal by cardiac macrophages (Nicolas-Avila et al., 2020; Zhang et al., 2023), and to mechanisms of protein sorting and loading into extracellular vesicles (e.g., exosomes) (Leidal et al., 2020). Besides degradative and secretory functions, autophagy has been implicated in the trafficking and function of RNAs. In particular, autophagy participates in intracellular and extracellular trafficking of RNA-binding proteins (e.g., HNRNPK. SAFB, MEX3A) and drives RNA sorting (particularly small non-coding RNAs, e.g., microRNAs) for nuclear enrichment or loading into extracellular vesicles (Leidal et al., 2020; Santovito et al., 2020). Notably, autophagy strongly influences microRNAs by affecting maturation through selective degradation of DICER and AGO2 (Gibbings et al., 2012; Liao et al., 2018), mediating intracellular and extracellular trafficking (Leidal et al., 2020; Santovito et al., 2020), ruling conventional and nonconventional functionality (Gibbings et al., 2012; Santovito et al., 2020; Santovito and Weber, 2022), and promoting their decay (Lan et al., 2014; Liao et al., 2018). Finally, ATGs are endowed with functions beyond membrane trafficking and contribute to signaling pathways independent of their role in autophagy, for example, by regulating p53-dependent apoptosis (ATG7) or the cGAS-STING pathway (BECLIN1, ATG9) with implications for cell cycle and innate immunity (Saitoh et al., 2009; Lee et al., 2012; Liang et al., 2014).

## 3 Autophagy in cardiac homeostasis and aging

Given the elevated energy demand (6 Kg ATP/day), it is not surprising that adequate mitochondrial function is crucial for cardiac activity and implies an essential role of autophagy as a mediator of mitochondrial quality control (Lopez-Crisosto et al., 2017). The first autophagosome detection by electron microscopy in fetal cardiomyocytes dates to the 1970s (Sybers et al., 1976). Since then, mechanistic studies in zebrafish and mice revealed that autophagy occurs during heart development during embryogenesis (assessed by the presence of LC3-GFP puncta) and that impairment of autophagy by constitutive deletion of ATGs (e.g., *Atg5*) determined aberrant expression of the cardiac patterning gene *Tbx2* with abnormalities of chamber septation and valve development (Lee et al., 2014). Consistently, Atg5-dependent autophagy is required for direct cardiac reprogramming that converts fibroblasts into contractile cardiomyocytes, and the fibroblast growth factor (FGF) signaling axis suppresses cardiomyocyte differentiation from mesodermal cells by inhibiting autophagy (Zhang et al., 2012; Wang et al., 2020). Surprisingly, Beclin1 (the mammalian *Atg6* orthologue, an autophagy activator) suppresses cardiomyocyte differentiation via an autophagy-independent mechanism (Wang et al., 2020). As a form of autophagy selective for dysfunctional mitochondria, mitophagy is mandatory for perinatal cardiac development, and *Park2* deletion to inhibit mitophagy results in lethal cardiomyopathy due to impaired disposal of fetal mitochondria, which must be replaced by adult ones after birth to switch substrate predilection from carbohydrates to fatty acids (Gong et al., 2015). Notably, disrupting the insulin/IGF1 signaling pathway (cardiomyocyte-specific *Irs1* and *Irs2* deletion) in neonatal mice resulted in unrestrained autophagy that paradoxically precipitated mitochondrial dysfunction and cardiomyocyte loss (Riehle et al., 2013). These findings unveil the complexity of autophagic signaling pathways and the importance of noncanonical functions of ATGs in cardiomyocyte differentiation and perinatal cardiac physiology.

Besides cardiac development, autophagy is crucial for the homeostasis of the adult heart. Conditional disruption of autophagy in cardiomyocytes (in *Mer* ^*Cre*^ *Mer,Atg5* ^*fl/fl*^ mice) leads to the accumulation of ubiquitinated elements, affecting sarcomere structure and mitochondrial alignment, favoring the development of contractile dysfunction and left ventricular dilatation (Nakai et al., 2007). Similarly, the genetic deletion of *Drp1*, which is required for mitophagy in cardiomyocytes, suppressed the autophagic flux with consequent left ventricular dysfunction and hypertrophy, eventually culminating in death within 13 weeks (Ikeda et al., 2015). Finally, the deletion of *Lamp2* in mice and humans (i.e., Danon disease) determines defective autophagic flux and affects CMA resulting in a vacuolar myopathy of cardiac and skeletal muscle with progressive cardiomyopathy which predominantly manifests a hypertrophic phenotype (Nishino et al., 2000; Tanaka et al., 2000; Maron et al., 2009). Together, these observations support the constitutive homeostatic role of autophagy and mitophagy in preserving cardiac structure and function.

With the increase in life expectancy, the attention to mechanisms of cardiac aging has substantially risen. Adult cardiomyocytes are terminally differentiated cells with limited proliferative capability, thus maintenance of their functional homeostasis is crucial for the aging heart. Autophagy declines with aging enhancing susceptibility to aging-associated cardiac dysfunction, and cardiac-specific disruption of autophagy (i.e., *Myh6* ^*Cre+*^ *;Atg5* ^*fl/fl*^) determined age-related cardiomyopathy (Taneike et al., 2010). Multiple mechanisms underlie the aging-related reduction of autophagic activity and involve signaling pathways implicated in longevity. In particular, aging is associated with lower AMPK activity and hyperactivation of the Akt and mTOR pathways, all able to dampen autophagy through inhibiting the ULK1 complex and deactivating (by phosphorylation) transcription factors involved in the transcription of ATGs, such as TFEB or Forkhead box O (FoxO) (Turdi et al., 2010; Kim et al., 2011; Martina et al., 2012; Roczniak-Ferguson et al., 2012; Saxton and Sabatini, 2017; Audesse et al., 2019). Moreover, the age-dependent decrease of nicotinamide adenine dinucleotide (NAD^+^) reduces SIRT1 activity and increases the acetylation of FoxO, ATG5, ATG7, and ATG8, thereby inhibiting their function to restrain autophagy (Lee et al., 2008; Khalil et al., 2016; Ren et al., 2017; Audesse et al., 2019). Aging also determines epigenetic changes to repress ATGs such as Dnmt2-dependent hypermethylation of *Atg5* and *Lc3b* promoters (Khalil et al., 2016). Besides dysregulation of signaling pathways, excessive accumulation of reactive oxygen species (ROS) and damaged mitochondria occurs with age and may reduce effective autophagy because of exhaustion of the autophagic machinery, oxidization and inactivation of ATGs (e.g., ATG3 and ATG7), and lipofuscin accumulation in lysosomes to impair their function (Brunk and Terman, 2002; Lee et al., 2008; Frudd et al., 2018). Furthermore, aging is associated with higher myocardial expression of inositol 1,4,5-trisphosphate receptors (IP3Rs) which are involved in the suppression of autophagy by assembling an inhibitory IP3R/BECLIN1 complex (Gorza et al., 1997; Decuypere et al., 2011; Wong et al., 2013). Finally, NLRP3 inflammasome activity is increased in aged hearts and preliminary evidence supports its possible contribution to cardiac aging via regulating autophagy (Miyamoto, 2019).

Aside from the evidence of reduced autophagy occurring in aging, mechanistic studies proved the opportunity of manipulating autophagy to improve age-induced cardiac dysfunction. Ubiquitous overexpression of *Atg5* in transgenic mice results in the activation of autophagy and significantly extends lifespan (Pyo et al., 2013). Moreover, transgenic expression of a mutated form of Beclin1 unable to interact with BCL2 in mice increased basal autophagy in the heart (and several other organs) and increased lifespan with reduced age-induced cardiac remodeling and lower spontaneous tumorigenesis (Fernandez et al., 2018). In line with the anti-aging effect of mitophagy, the genetic deficiency of Parkin in mice is associated with age-dependent accumulation of dysfunctional mitochondria in the heart (Kubli et al., 2013), whereas transgenic overexpression of Parkin reduced aging-induced cardiac damage and preserved cardiac function by reducing the accumulation of dysfunctional mitochondria, ROS generation, and inflammaging biomarkers (e.g., senescence-associated β-galactosidase) (Hoshino et al., 2013). Finally, autophagy is instrumental in cardioprotective and anti-aging effects of oral administration of spermidine as well as in the improved myocardial bioenergetics and age-related dysfunction upon IGF1R inhibition in aged mice (Eisenberg et al., 2016; Abdellatif et al., 2022). Altogether, strong evidence supports the role of basal autophagy in maintaining cardiac homeostasis and highlights the potential benefit of its manipulation to revert age-induced cardiac dysfunction.

## 4 Autophagy in cardiomyocyte ischemic response

Both ischemia and reperfusion trigger autophagy in the myocardium, although the causative role of autophagy in ischemia-reperfusion (I/R) injury is still not entirely understood. A reason for this is that some conflicting results exist in the literature, as described in the following. During myocardial ischemia in mice, nutrient deprivation leads to the autophagy activation, which is accompanied by activation of AMPK and inhibited by dominant negative AMPK (Matsui et al., 2007). Likewise, glucose deprivation in cultured cardiac myocytes induces AMPK activation and autophagy, while inactivating mTOR. This can be considered an adaptive mechanism for maintaining ATP production by generating free fatty acids (FFAs) and amino acids (Kanamori et al., 2011). Furthermore, the disposal of damaged mitochondria helps preventing cardiomyocyte damage and apoptosis (Yan et al., 2005). In support of this, disruption of dynamin-related protein 1 (Drp1), a GTPase that mediates mitochondrial fission, inhibited mitochondrial autophagy, and resulted in mitochondrial dysfunction, thereby promoting cardiac dysfunction and increased susceptibility to I/R (Ikeda et al., 2015). An alternative pathway for mitophagy utilizes the serine/threonine protein kinase Unc-51-like kinase 1 (Ulk1) and the small GTPase Rab9 to clear damaged mitochondria independently of conventional autophagy proteins. Ulk1 phosphorylation of Rab9 at serine 179 is critical for alternative mitophagy and cardioprotection during ischemia (Saito et al., 2019).

Furthermore, the small GTP-binding protein Rheb activates the complex 1 of the mechanistic target of rapamycin (mTORC1) and has been identified as a critical regulator of autophagy during cardiac ischemia, in the setting of metabolic disease (Sciarretta et al., 2012). Mice subjected to a high fat diet had a disturbed, uncontrolled activation of the Rheb/mTORC1 pathway which leads to autophagy inhibition and a reduction of myocardial tolerance to ischemia. Another key factor involved in cardiomyocyte autophagy induction is NADPH oxidase (Nox) 4, an enzyme that generates ROS during energy stress in the heart, thereby preserving cellular energy and limiting cell death in energy-deprived cardiomyocytes (Sciarretta et al., 2013).

Autophagy during the reperfusion phase is also triggered by oxidative stress, but linked to an upregulation of Beclin 1 and considered detrimental in this condition according to some studies (Valentim et al., 2006; Matsui et al., 2007; Hariharan et al., 2011). In rat cardiomyocytes, the knockdown of Beclin 1 expression by RNA interference inhibited autophagy, while enhancing cell survival (Valentim et al., 2006). Beclin 1 plays a key role at the interface between autophagy and apoptosis, which are tightly connected cellular processes (Kang et al., 2011). The proapoptotic kinase mammalian Ste20-like kinase-1 (Mst-1) acts as a molecular switch that selectively drives autophagy or apoptosis by preferentially altering the formation of Bcl-2–Beclin 1 complexes (Maejima et al., 2013). In various transgenic mouse models subjected to permanent left anterior descending (LAD) ligation, Mst1 promoted cardiac dysfunction by inhibiting autophagy, associated with increased levels of Thr_108_-phosphorylated Beclin1. Mechanistically, activation of Mst-1 in energy-deprived cardiomyocytes resulted in Beclin1 phosphorylation, which enhanced the interaction between Beclin1 and Bcl-2 and Bcl-xL.

Impaired autophagic flux during reperfusion may represent a pathological mechanism contributing to cardiomyocyte death. In support of this, reoxygenation of rat neonatal cardiomyocytes after hypoxia increased cell death compared with hypoxia alone, which was accompanied by markedly increased autophagosomes but not autolysosomes, and impaired clearance of polyglutamine aggregates, indicating a disturbed autophagic flux (Ma et al., 2012). This defect was linked to a reduced expression of LAMP2, a critical determinant of autophagosome-lysosome fusion. The resulting autophagosome accumulation was associated with increased ROS and ROS-induced BECLIN1 upregulation, mitochondrial permeabilization, and cardiomyocyte death. Phosphorylation of the mitochondrial serine/threonine kinase beta isoform-specific glycogen synthase kinase-3 (GSK-3) is a central downstream event of multiple cardioprotective pathways. Inhibition of GSK-3β stimulated mTOR signaling and inhibited autophagy through a rapamycin-sensitive (mTOR dependent) mechanism (Zhai et al., 2011). Using gain- and loss-off-function transgenic mouse models, GSK-3β signaling was shown to exert distinct effects on cardiac injury caused by either 2 hours of non-reperfused ischemia or I/R, indicating that isoform-specific inhibition of GSK-3β exacerbates ischemic injury but protects against I/R injury by modulating mTOR and autophagy.

As opposed to the above-described evidence for impaired autophagy or detrimental effects of autophagy in I/R injury, other studies reported cardioprotective effects of autophagy induction in this condition. Autophagy induction with rapamycin dampened I/R injury in a mechanism involving JAK2-STAT3 signaling in cardiomyocytes (Das et al., 2012). Likewise, treatment with FDA-approved HDAC inhibitors for cancer treatment was shown to limit myocardial I/R damage through autophagy and mitochondrial biogenesis, thereby preserving mitochondrial homeostasis in cardiomyocytes (Xie et al., 2014; Yang et al., 2019). Furthermore, recent findings have shown that atrial natriuretic peptide (NPPA) mediates cardioprotection against I/R injury by activating autophagy in cardiomyocytes through NPR1/type A natriuretic peptide receptor and PRKG/protein kinase G signaling (Forte et al., 2022). Interestingly, NPPA produced by cardiomyocytes is secreted in response to energy deprivation or hypoxia, thereby providing an autocrine/paracrine stimulus for autophagy induction. This may provide a mechanistic explanation for the well-established cardioprotective and anti-hypertrophic effects of NPPA in cardiac stress conditions.

In summary, the precise cellular survival or cell death pathways in cardiomyocytes seem highly context-dependent. Autophagy during myocardial I/R injury appears as a double-edged sword, which may initially represent a cellular quality control and prosurvival mechanism, but eventually turns into a detrimental process. This might be linked to disturbed autophagosome degradation. The precise mechanisms and conditions that may turn into a detrimental outcome deserve further investigation, in view of potential therapeutic strategies to improve cardioprotective autophagic flux in the acute phase post-MI. Some preclinical data have indeed shown the beneficial effects of autophagy induction by limiting adverse remodeling in permanent LAD ligation models, e.g., by treatment with trehalose (Sciarretta et al., 2018). Moreover, targeting particular long noncoding RNAs to modulate autophagy has been suggested as a therapeutic strategy in the context of myocardial infarction and heart failure (Wang et al., 2015; Liu et al., 2018; Liang et al., 2020).

## 5 Autophagy orchestrates immune cell function and response to cardiac injury and ischemia

While the previous section focused on the role of autophagy in cardiomyocytes during cardiac ischemic stress and reperfusion injury, it is also important to consider the role of innate immune cells in this context. Here, we will focus on mechanisms that are relevant for maintaining cardiac homeostasis in steady-state conditions, as well as in post-myocardial infarction inflammation and subsequent repair processes.

To maintain the high cardiac energy demand required for contraction and relaxation, cardiomyocytes contain a large number of mitochondria. The maintenance of a pool of healthy mitochondria is essential for sustaining normal cardiac performance. Therefore, mitochondrial recycling and quality control are tightly controlled via mitophagy. However, given that cardiomyocytes are subjected to intense mechanical stress and metabolic demands, the question arises of how these postmitotic cells with virtually no turnover are able to preserve cellular homeostasis. In a recent study, Nicolas-Avila et al. (2020) describe a new mechanism of autophagic-driven mitochondria release from cardiomyocytes to MerTK^+^ macrophages. Advanced imaging techniques including light sheet microscopy and confocal microscopy revealed that cardiomyocytes are surrounded by on average five cardiac macrophages and form direct interactions. Cardiomyocytes thereby eject dysfunctional mitochondria and other cargo through an autophagosomal process into particles called exosphers. The uptake of these exosphers by the surrounding macrophages is mediated by Mertk and prevents extracellular waste accumulation and inflammasome activation, which is crucial for cardiac homeostasis. Ischemic cardiac stress by permanent LAD ligation increased mitochondrial ejection via exosphers.

Another important study highlighted the contribution of autophagy in the maturation and function of neutrophils (Riffelmacher et al., 2017). Neutrophils play a critical role as one of the first lines of innate immune response to protect the host from exogenous pathogens and to repair the damaged tissue. Due to their short lifespan, neutrophils are constantly produced in the bone marrow from hematopoietic progenitors in a process named granulopoiesis and released as mature neutrophils into the blood stream (Soehnlein et al., 2017). Riffelmacher et al. (2017) found that the generation of FFAs via autophagy is essential for neutrophil differentiation. The highest levels of autophagic flux were observed in the early stages of differentiation, as compared to reduced autophagic flux observed at the final maturation stage. Targeted deletion of *Atg7* in neutrophil progenitors resulted in an accumulation of immature neutrophils in the bone marrow. More detailed metabolic analyses further revealed that neutrophil differentiation was accompanied by a shift towards mitochondrial respiration with the downregulation of glycolysis, which was blunted in autophagy-deficient neutrophils. Adding exogenous FFAs to *Atg7*-deficient neutrophil precursors restored their differentiation, suggesting that the oxidation of FFAs produced by autophagy provides the necessary ATP for neutrophil differentiation. A different study demonstrated that autophagy is also required for neutrophil degranulation and NADPH-oxidase-mediated reactive oxygen species production (Bhattacharya et al., 2015). Myeloid-specific deletion of Atg7 reduced the inflammatory activity of neutrophils *in vitro* and in a murine model of experimental autoimmune encephalomyelitis. Given the important role of neutrophils in acute myocardial I/R injury, chronic ischemia, and remodeling (Horckmans et al., 2017; Puhl and Steffens, 2019), it is likely that neutrophil autophagic flux may also be a critical regulator of cardiac injury and remodeling. In particular, the relevance of neutrophil-secreted factors (including neutrophil gelatinase-associated lipocalin, NGAL) in the regulation of macrophage polarization during the post-ischemic myocardial healing phase was demonstrated in an experimental model of permanent infarction (Horckmans et al., 2017). A more recent study highlighted the relevance for endothelial autophagy in regulating neutrophil infiltration to sites of inflammation (Reglero-Real et al., 2021). Inflamed venular endothelial cells upregulated autophagy selectively at endothelial cell junctions, which was temporally aligned with the peak of neutrophil trafficking. Endothelial cell *Atg5* deficiency resulted in excessive neutrophil transendothelial migration and uncontrolled leukocyte migration in murine inflammatory models, while pharmacological induction of autophagy suppressed neutrophil infiltration into tissues. However, the precise mechanisms of neutrophil extravasation in the ischemic myocardium have not been studied as imaging of leukocyte trafficking in the beating heart remains very challenging (Steffens et al., 2022). Hence, we may only speculate that the molecular mechanisms determining neutrophil diapedesis in remote areas of the infarcted heart or reperfused infarct zone are comparable to the processes studied in venules of other inflammatory sites.

In addition, there is evidence that autophagy is relevant for monocyte differentiation into macrophages (Jacquel et al., 2012), a process orchestrated by colony-stimulating factor-1 (Pittet et al., 2014). While the healthy heart contains a heterogeneous population of tissue-resident macrophages with distinct origins and functions, the macrophage repertoire becomes even more diverse in response to cardiac injury, as blood-borne monocytes migrate into the myocardium and differentiate into macrophages to remove dying tissue, scavenge pathogens and promote healing (Swirski and Nahrendorf, 2018; Zaman and Epelman, 2022). Hence, it can be speculated that monocyte autophagic flux is an important process in the immune response to cardiac injury. However, an experimental study focusing on macrophage lysosomal function in post-myocardial infarction adverse remodeling found that ATG-dependent autophagy was dispensable, at least in this particular experimental model (Javaheri et al., 2019). Inducible macrophage-specific overexpression of transcription factor EB (TFEB), a master regulator of lysosome biogenesis, attenuated post-I/R cardiac remodeling and decreased the abundance of pro-inflammatory macrophages. Surprisingly, all these effects were independent of myeloid ATG5 expression. However, given that this study only focused on a single time point after 4 weeks of I/R injury, a more in-depth investigation is warranted to clarify the role of monocyte-macrophage autophagy in cardiac injury responses.

In summary, it will be interesting to investigate how myocardial infarction and I/R injury affect endothelial, neutrophil and monocyte autophagic flux, which may have crucial implications on emergency granulopoiesis, cardiac neutrophil infiltration, degranulation, ROS production, and cardiac macrophage phenotypes. Consequently, pharmacological targeting of autophagy in this condition could represent a possible therapeutic strategy to limit MI-induced cardiac damage, adverse remodeling and heart failure.

## 6 Autophagy adaptor protein in cardiac aging and ischemia

Evidence on the contribution of selective autophagy in cardiac pathophysiology is accumulating and extends beyond mitophagy (Kirkin et al., 2009). A crucial role in selectivity is exerted by adaptor proteins that bind specific cargoes and interact with conjugated LC3 via conserved LIR domains. The proteins SQSTM1 (also known as p62) and Neighbor of BRCA1 gene 1 (NBR1) are among the best-characterized examples (Gatica et al., 2018; Rasmussen et al., 2022). Structurally, they own an LIR motif, homo- or hetero-oligomerization domains, and a C-terminal ubiquitin-binding (UBA) domain binding ubiquitinated cargos. In clearing misfolded proteins, polyubiquitination of the cargo is essential so that they can bind to SQSTM1, be included in autophagosomes, and then be sent for lysosomal degradation (Gatica et al., 2018). The UBA domain interacts with ubiquitin chains attached to the cargo, while the LIR motif interacts with ATG8 family proteins (e.g., LC3-II) attached to the inner membrane surface of a growing phagophore, which then closes to become an autophagosome (Lamark et al., 2017). SQSTM1 tends to cluster in p62 bodies when its levels are increased. In human cells, p62 bodies are often observed as discrete punctae. The formation of p62 bodies depends on the N-terminal Phox/Bem1p (PB1) domain-mediated polymerization of SQSTM1 and is mediated by NBR1 (Lamark and Johansen, 2021). NBR1 serves as a chain terminator of SQSTM1 filaments (Jakobi et al., 2020). Since shorter SQSTM1 filaments form p62 bodies more easily, NBR1 plays a key role in promoting their assembly by regulating p62 filament length. Furthermore, NBR1 contains multiple domains involved in cargo recruitment, and the interaction between the SQSTM1 and NBR1 allows for more efficient cargo recognition. Given the strong cooperative activities of NBR1 and SQSTM1, it is often difficult to distinguish the specific effects of each protein. A reduction in SQSTM1 levels, autophagosomes, and p62 bodies correlates with the progression of the autophagic flux toward its late steps. Their amount is inversely related to cellular autophagy levels, decreasing when lysosomal degradation has occurred successfully (Katsuragi et al., 2015).

Notably, upregulation of SQSTM1 has been observed in most human failing hearts due to ischemic and non-ischemic heart disease (Weekes et al., 2003; Sanbe et al., 2005), implying that the accumulation of misfolded/damaged proteins (i.e., increased proteotoxic stress) due to blockage of selective autophagy is likely a common pathogenic feature for the progression of a large subset of heart disease to congestive heart failure. In the example, desmin-related cardiomyopathy is determined by the accumulation of desmin-misfolded aggregates and is characterized by higher expression of SQSTM1 at mRNA and protein levels and *SQSTM1* silencing impaired autophagosomal formation, exacerbated cell injury, thus increasing cardiomyocyte death (Zheng et al., 2011). Aging is also characterized by the accumulation of protein aggregates. Analysis of human specimens from young (10 years old) and aged individuals (65 years old) revealed a significantly higher accumulation of SQSTM1 in aged hearts with a direct correlation with age (Li C. et al., 2020). Loss of SQSTM1 has been associated with accelerated aging, while overexpression of the SQSTM1 and NBR1 in *Drosophila* and *C. Elegans* increases lifespan suggesting their possible protective role during aging (Aparicio et al., 2019; Kumsta et al., 2019). In line with a protective role against proteotoxic stress, SQSTM1 is required to increase the autophagic flux in cardiomyocytes with dysfunctional proteasomal degradation of ubiquitinylated proteins, and its genetic deletion aggravated diastolic dysfunction upon pharmacological inhibition of the proteasome (Pan et al., 2020). On the other hand, in the context of ischemia-reperfusion, SQSTM1 forms a complex with the necrosome proteins RIP1 and RIP3, and its silencing *in vivo* protects the aged hearts from necrosis (Li C. et al., 2020). Similarly, SQSTM1 may scaffold other proteins involved in cell death mechanisms, such as caspases, and is instrumental to the homocysteine-induced apoptosis and autosis (an autophagy-dependent cell death) of cardiomyocytes (Yin et al., 2022). These findings further highlight the complexity of autophagy and its mechanisms in cardiac biology.

In conclusion, selective autophagy uses SQSTM1 as critical receptors for cargo selection in cooperation with NBR1 and other adaptors. Disruptions in their ability to deliver specific cargo for degradation may lead to disruption of cell signaling homeostasis with important implications for several cardiovascular diseases.

## 7 Targeting autophagy in non-ischemic cardiac diseases

Under normal conditions, the myocardium has low levels of basal autophagy. Stress conditions can increase its levels to enhance cell survival, with constitutive autophagy maintaining normal cardiac structure and function, and upregulated autophagy occurring during cardiac disease (Rothermel and Hill, 2008; Gustafsson and Gottlieb, 2009). However, in several cardiac diseases, autophagy can be downregulated or hyperactivated, therefore becoming detrimental. Patients with congestive heart failure (Takemura et al., 2006), coronary artery disease (Yan et al., 2005), arterial hypertension, hypertrophy and aortic valvular disease (Nakai et al., 2007), diabetic cardiomyopathy, and cardiac senescence display increased autophagosomal accumulation in myocardial biopsies (Terman and Brunk, 2005; Shinmura et al., 2011). In cardiac hypertrophy (Hill and Olson, 2008), autophagy plays a role in the progression of structural remodeling toward heart failure (Hein et al., 2003; Zhu et al., 2007). In heart failure, increased autophagy can cause cardiac dysfunction, with autophagy-induced degeneration leading to cardiac cell death (Akazawa et al., 2004; Schiattarella and Hill, 2016). Several treatments have been shown to regulate (either inducing or inhibiting) cardiac autophagy, including drugs for the treatment of cardiovascular diseases (e.g., verapamil, amiodarone, metoprolol), diabetes (e.g., metformin), and anti-neoplastic drugs (e.g., doxorubicin) (Table 1). There are scant data on the downregulation of autophagy in anthracycline-induced cardiotoxicity (Ma et al., 2017; Li M. et al., 2020; Wang et al., 2021), while no data are available on autophagy and tyrosine kinase inhibitors (TKI)-induced cardiotoxicity. The activating or inhibiting effects of TKI on autophagy largely depend on the cell type (Hirschbuhl et al., 2021). We were the first to demonstrate that ponatinib, the most cardiotoxic agent amongst all FDA-approved TKIs in the treatment of chronic myeloid leukemia, decreased autophagosome formation as well as LC3-II and p62 expression in cardiomyocytes, indicating a blockage of autophagic flux (Madonna et al. Eur Heart J Suppl abstract in Frontiers in CardioVascular Biomedicine 2022). Taken together, these data suggest that autophagy may represent a valuable target for limiting damage in non-ischemic cardiac diseases. However, despite the existence of an intimate connection between autophagy and the heart, only a few selective autophagy activator candidates have been recognized so far, depending on the context of cardiac homeostasis and disease.

**TABLE 1:** Autophagy-inducing or inhibiting drugs in cardiovascular disease.

Drug	Current use	Autophagy	References
Amiodarone	Arrythmia	Activates	Balgi et al. (2009)
Bortezomib	Myeloma, lymphoma	Activates	Wu et al. (2020)
Clonidine	Hypertension	Activates	Williams et al. (2008)
Digoxin	Arrythmia	Activates	Skubnik et al. (2021)
Doxorubicin	Solid cancer	Activates (early phase), inhibits (late phase)	Wang et al. (2021)
Dronedarone	Arrythmia	Activates	Piccoli et al. (2011)
Isoprotenerol, norepinephrine	Bradycardia, hypotension	Activates	Lu et al. (2016)
Ivabradine	Arrythmia	Activates	Dai et al. (2021)
Metformin	Diabetes	Activates	Meley et al. (2006)
Metoprolol, propanolol	Arrythmia, hypertension	Activates	Sveshnikov et al. (2011)
Nifedipine	Hypertension	Activates	Pushparaj et al. (2015)
Olmesartan	Hypertension	Activates	Sukumaran et al. (2011)
Paclitaxel	Stent restenosis	Inhibits	Hayashi et al. (2009)
Ranolazine	Stable angina, arrhytmia	Activates	Guerra et al. (2017)
Rapamycin	Preventing cardiac transplantation rejection, stent restenosis	Activates	Soderlund and Radegran (2015)
iSGLT2	Diabetes, heart failure	Activates or inhibits	Packer (2020); Madonna et al. (2023)
Statins	Cholesterol lowering	Activates	Andres et al. (2014)
Tamoxifen	Breast cancer	Activates	Kocaturk et al. (2019)
Verapamil	Hypertension, unstable angina, arrythmia	Activates	Zhang et al. (2007)

Legend: iSGLT2, Sodium-glucose cotransporter 2 inhibitor.

Under stress conditions, such as starvation, autophagy is increased in the heart, and FYVE And Coiled-Coil Domain Autophagy Adaptor 1 (FYCO1) has been linked to autophagy. FYCO1 is highly expressed in the heart and its role has recently been investigated *in vitro and in vivo* under basal and stress conditions (Kuhn et al., 2021). FYCO1 directly interacts with LC3, Rab7, and phosphatidylinositol-3-phosphate (PI3K), key players in autophagy (Pankiv et al., 2010). Although FYCO1 knockdown reduces autophagy in isolated rat cardiomyocytes, overexpression of FYCO1 leads to increased autophagic flux *in vitro*. Since overexpression of FYCO1 prevents cardiac dysfunction in response to biomechanical stress, enhancing autophagic flux by overexpressing FYCO1 could be a promising therapeutic strategy to treat or prevent heart failure (Kuhn et al., 2021).

In adult mice, cardiac-specific deletion of *Atg5* leads to contractile dysfunction, hypertrophy and cardiomyopathy, consistent with the notion that basal autophagy levels in cardiomyocytes are required for cellular proteostasis (Nakai et al., 2007). *In vitro* studies with cardiomyocytes harvested from *Atg5*-deficient mice revealed that deficiency of the autophagic gene could cause the accumulation of unwanted proteins and contribute to myocardial disease (Pattison and Robbins, 2011). In line with these findings, *Lamp2*-deficient mice displayed increased autophagic vacuole accumulation and could not degrade proteins, thereby promoting cardiomyopathy (Nishino et al., 2000; Tanaka et al., 2000; Maron et al., 2009).

In the failing heart, autophagy can cause myocardial cell damage via PARP1 (Poly ADP ribose polymerase), which promotes autophagy in cardiomyocytes by modulating FoxO3a (a member of the FoxO family of transcription factors) (Schiattarella and Hill, 2016). Changes in cardiac autophagy during sepsis have not been clearly defined. BECLIN1, an early effector of autophagy in mammals, is ubiquitously expressed (Liang et al., 1998; Liang et al., 1999). Autophagy changes in response to sepsis severity, and Beclin 1 plays a key role in the autophagic response of the septic heart in a mouse model of LPS-induced sepsis (Sun et al., 2018). Previous preclinical studies evaluating autophagy as a therapeutic approach for sepsis were mainly focused on the mTOR inhibitor rapamycin (Hsieh et al., 2011). Because mTOR is involved in the regulation of a variety of pathways, rapamycin may cause unwanted toxicity. In this regard, Sun et al. (2018) showed that forced overexpression of Beclin 1 in the heart promotes autophagy and mitophagy, protects mitochondria, and improves cardiac function, suggesting Beclin 1 as a better therapeutic approach to modulate autophagy. In Beclin 1 haploinsufficient mice, load-induced increases in autophagy activity were blunted, and pathological remodeling of the left ventricle was moderately diminished. In contrast, in mice engineered for forced overexpression of Beclin 1 in cardiomyocytes (MHC-beclin-1), pressure overload triggered an amplified autophagic response and pathological remodeling of the heart was more severe (Sun et al., 2018). In 2009, researchers identified Rubicon as a protein that suppresses autophagy by interacting with the Beclin 1 complex (Matsunaga et al., 2009; Zhong et al., 2009). Cardiomyocyte-specific conditional knockout of Rubicon restores autophagic flux and reduces the rate of autotic cell death rate injury in the heart during the late phase of ischemia/reperfusion injury in the heart (Nah et al., 2020). In mouse models, Rubicon deficiency enhances autophagic flux in the heart during LPS-induced sepsis, thereby maintaining cardiac stroke volume but without affecting myocardial inflammatory responses (Zi et al., 2015). Thus, targeting Rubicon may be a promising modality of autophagy modulation in various cardiac conditions.

Kanamori et al. (2015) examined possible differences in the autophagy process in animal models of type 1 and type 2 diabetes. While cardiac autophagic activity is enhanced in type 1, it is suppressed in type 2 diabetes. Lysosomes and autophagosomes accumulate within cardiomyocytes of type 1 diabetic mice, whereas abundant lipid droplets and immature autophagosomes were observed in the heart of type 2 diabetic mice (Kanamori et al., 2015). Here, resveratrol, an autophagy enhancer, mitigated diastolic dysfunction in the heart of type 2 diabetic mice, whereas it had opposite effects in the hearts of type 1 diabetic mice, suggesting that resveratrol may be a useful therapeutic target in diabetic cardiomyopathy, depending on the diabetic context (Kanamori et al., 2015). Similarly, resveratrol had beneficial effects in ischemic heart failure through autophagic activation (Kanamori et al., 2013). Consistent with the notion that insulin exerts an inhibitory effect on autophagy, our research group recently demonstrated hyperactivation of autophagy associated with left ventricular dysfunction, remodeling, fibrosis, and myocyte apoptosis in a murine model of insulin-deficient diabetic cardiomyopathy (Madonna et al., 2023). Here, empagliflozin preserved cardiac dysfunction and remodeling at least in part, through the inhibition of autophagy. This process was mediated by inactivating the autophagy inducer GSK3β, which resulted in increased serum response factor (SRF) interaction with serum response element (SRE) and subsequent upregulation of cardiac actin expression. Our results describe a novel paradigm in which empagliflozin inhibits the hyperactivation of autophagy through the GSK-3β signaling pathway in the context of diabetes.

**FIGURE 2:**
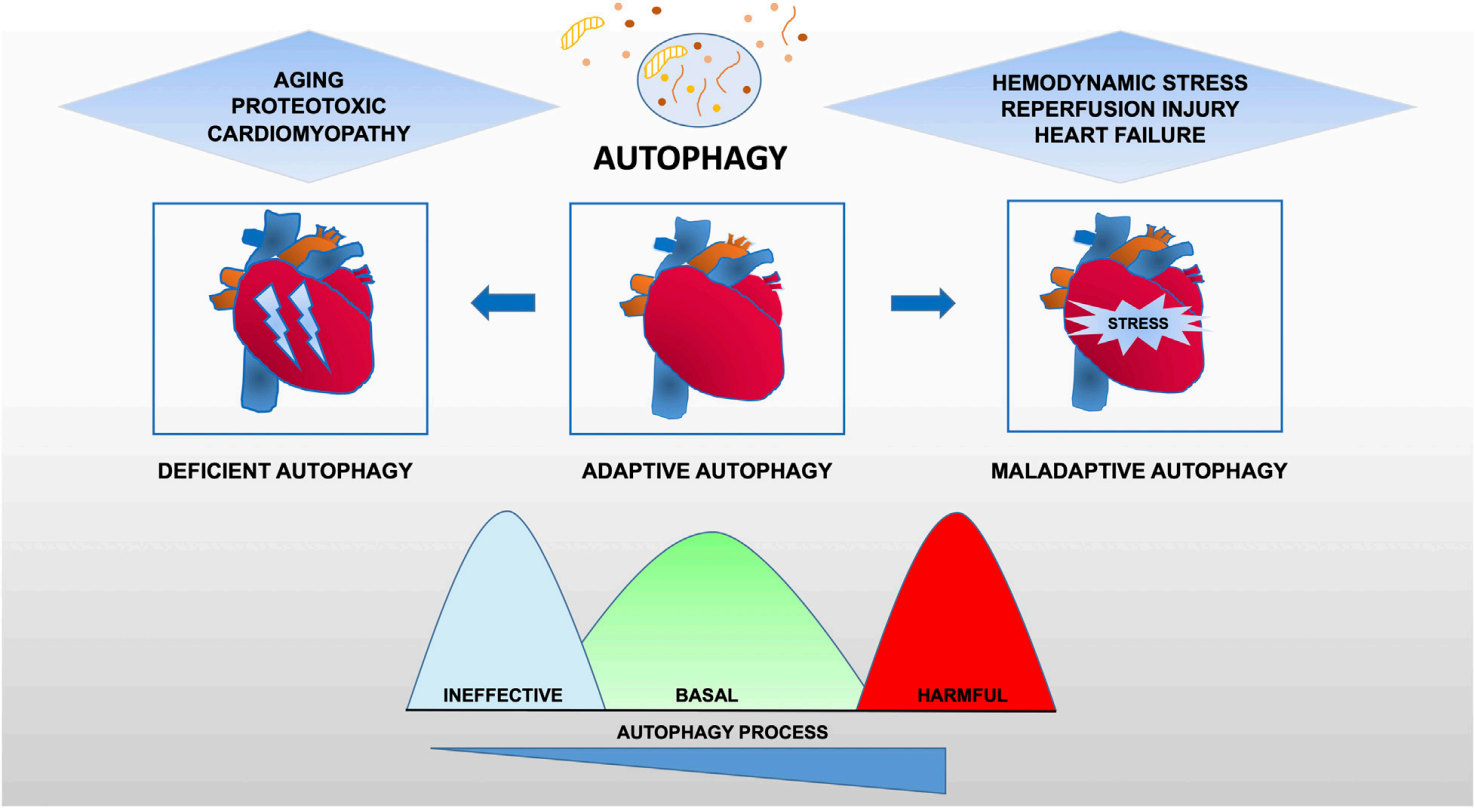
Functional relevance of autophagy for cardiac pathophysiology. Constitutive autophagy of cardiomyocytes under basal conditions is a homeostatic mechanism for normal cardiac structure and function. Autophagic activity is reduced during aging or following exposure to stressors such as chemotherapy drugs owning cardiotoxicity. However, in hearts exposed to hemodynamic overload or ischemia-reperfusion injury, autophagic activity is upregulated at supraphysiological levels, suggesting a contribution to the maladaptive response of the heart that may lead to heart failure.

Taken together, these data provide evidence for the maladaptive role of autophagy in cardiovascular disease, suggesting that there is an optimal zone of cardiomyocyte autophagy that may be beneficial and that treatments resulting in levels of autophagy outside (higher or lower) this therapeutic window are likely to be deleterious (Figure 2).

## 8 Conclusion

Compelling evidence revealed the crucial role of autophagy in preserving cardiac health by ruling cardiac homeostasis under baseline conditions and participating in the mechanisms of response to pathological injuries. While enhancing autophagy activation has shown beneficial outcomes in aging and longevity in animal models, the identification of a proper therapeutic window in diseased conditions and well-tolerated pro-autophagic drugs is an active research area. Meeting these medical needs will provide novel therapeutics and ultimately improve the outcome of patients with heart disease.
